# Supplementary figures and images for: Explaining conflict violence in terms of conflict actor dynamics
Source: Sci Rep. 2023 Dec 1;13:21187. doi: 10.1038/s41598-023-48218-x (PMC10692117; doi:10.1038/s41598-023-48218-x)

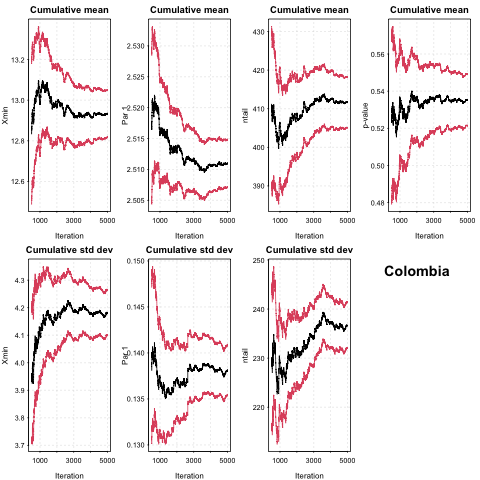

Supplement: Supplementary file 1 — Supplementary Information. [file 41598_2023_48218_MOESM1_ESM.zip › Explaining_conflict_violence_in_terms_of_conflict_actor_dynamics_/figs/colombia-bootstrapping-results.png]

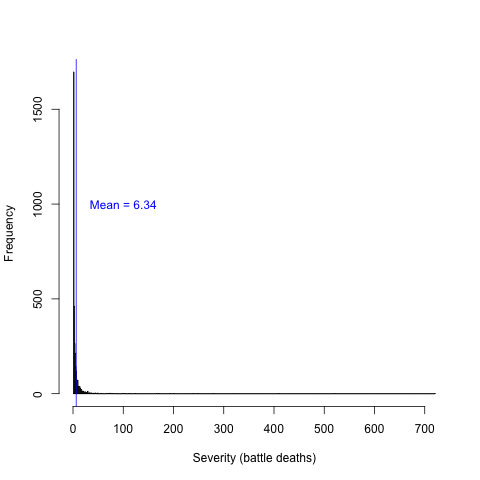

Supplement: Supplementary file 1 — Supplementary Information. [file 41598_2023_48218_MOESM1_ESM.zip › Explaining_conflict_violence_in_terms_of_conflict_actor_dynamics_/figs/colombia-frequency-distribution.png]

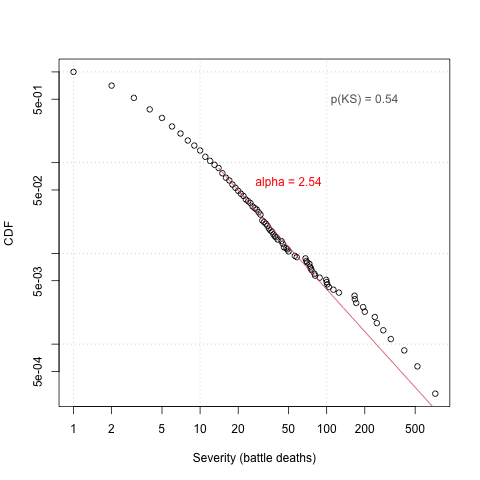

Supplement: Supplementary file 1 — Supplementary Information. [file 41598_2023_48218_MOESM1_ESM.zip › Explaining_conflict_violence_in_terms_of_conflict_actor_dynamics_/figs/colombia-frequency-size-distribution.png]

# Colombia departments, 1989–1999: Alpha

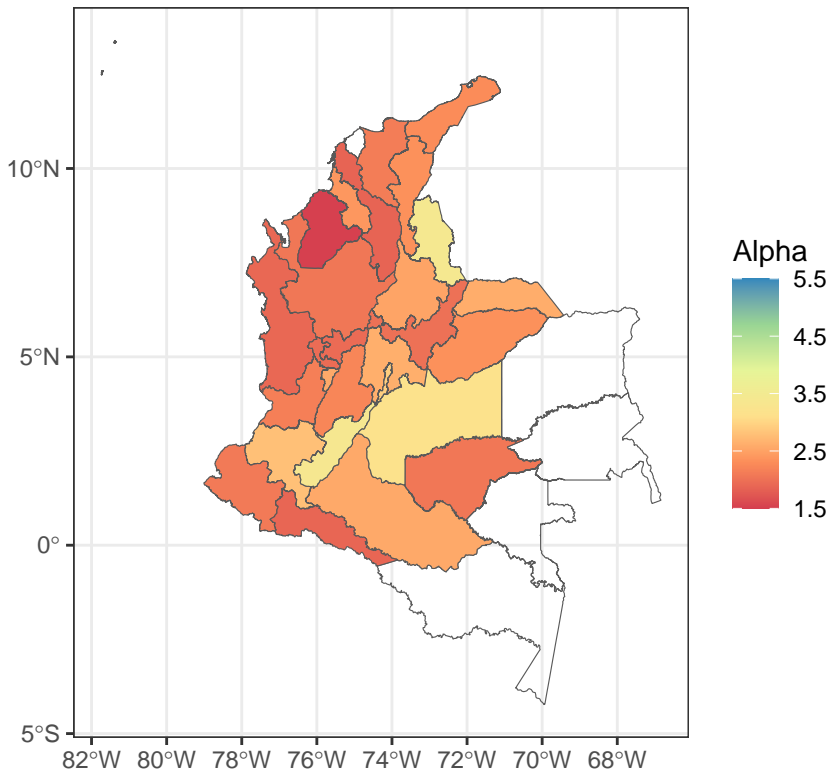

Supplement: Supplementary file 1 — Supplementary Information. [file 41598_2023_48218_MOESM1_ESM.zip › Explaining_conflict_violence_in_terms_of_conflict_actor_dynamics_/figs/department-1989-1999-alpha.pdf]

# Colombia departments, 1989–2018: Alpha

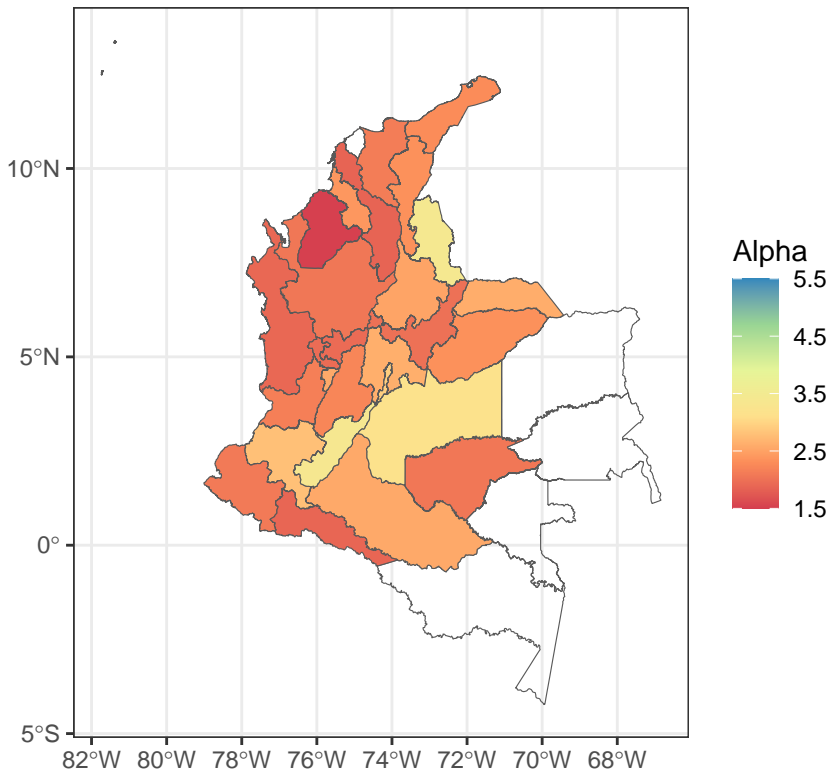

Supplement: Supplementary file 1 — Supplementary Information. [file 41598_2023_48218_MOESM1_ESM.zip › Explaining_conflict_violence_in_terms_of_conflict_actor_dynamics_/figs/department-1989-2018-alpha.pdf]

# Colombia departments, 2000–2009: Alpha

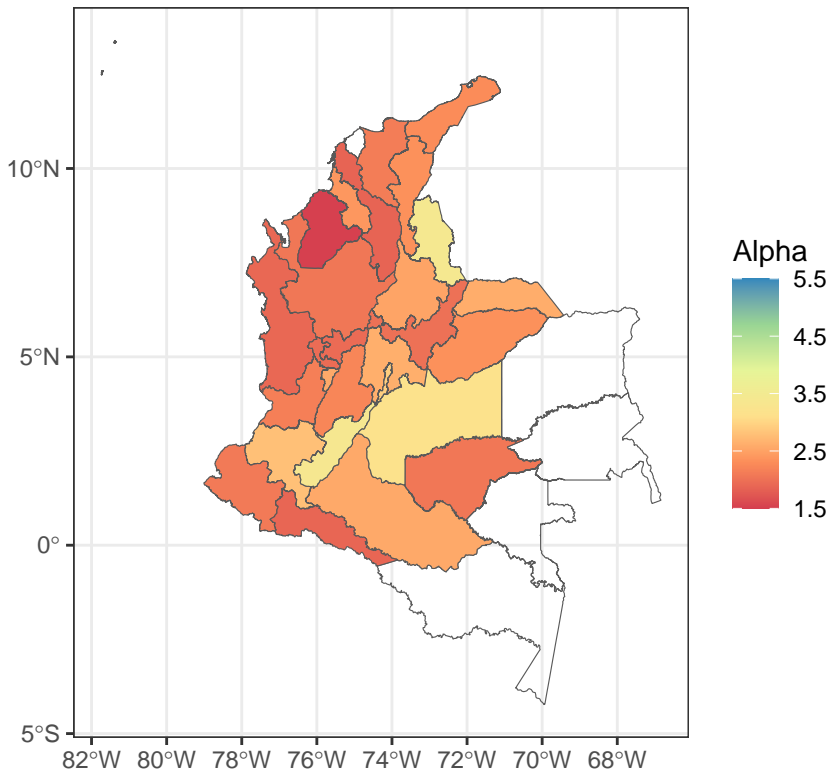

Supplement: Supplementary file 1 — Supplementary Information. [file 41598_2023_48218_MOESM1_ESM.zip › Explaining_conflict_violence_in_terms_of_conflict_actor_dynamics_/figs/department-2000-2009-alpha.pdf]

# Colombia departments, 2010–2018: Alpha

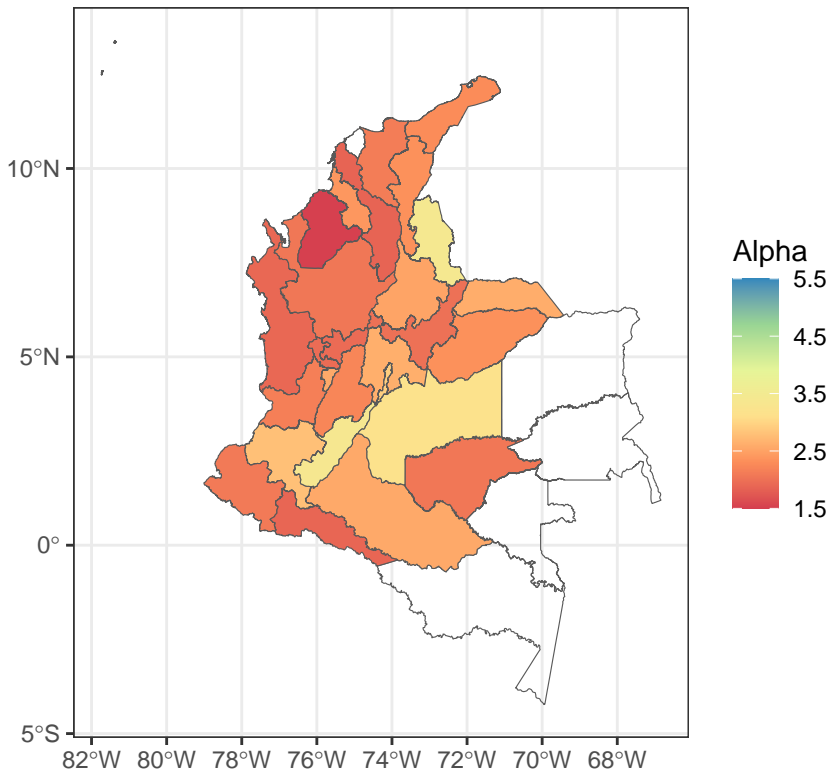

Supplement: Supplementary file 1 — Supplementary Information. [file 41598_2023_48218_MOESM1_ESM.zip › Explaining_conflict_violence_in_terms_of_conflict_actor_dynamics_/figs/department-2010-2018-alpha.pdf]

# Colombia departments, 1989–1999: P-value

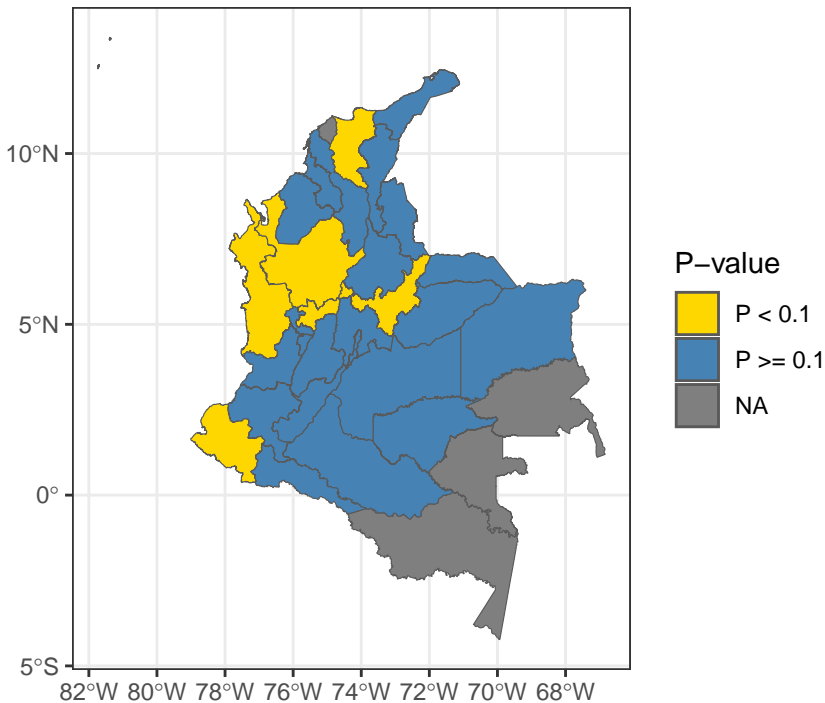

Supplement: Supplementary file 1 — Supplementary Information. [file 41598_2023_48218_MOESM1_ESM.zip › Explaining_conflict_violence_in_terms_of_conflict_actor_dynamics_/figs/departments-1989-1999-p-values.pdf]

# Colombia departments, 1989–2018: P-value

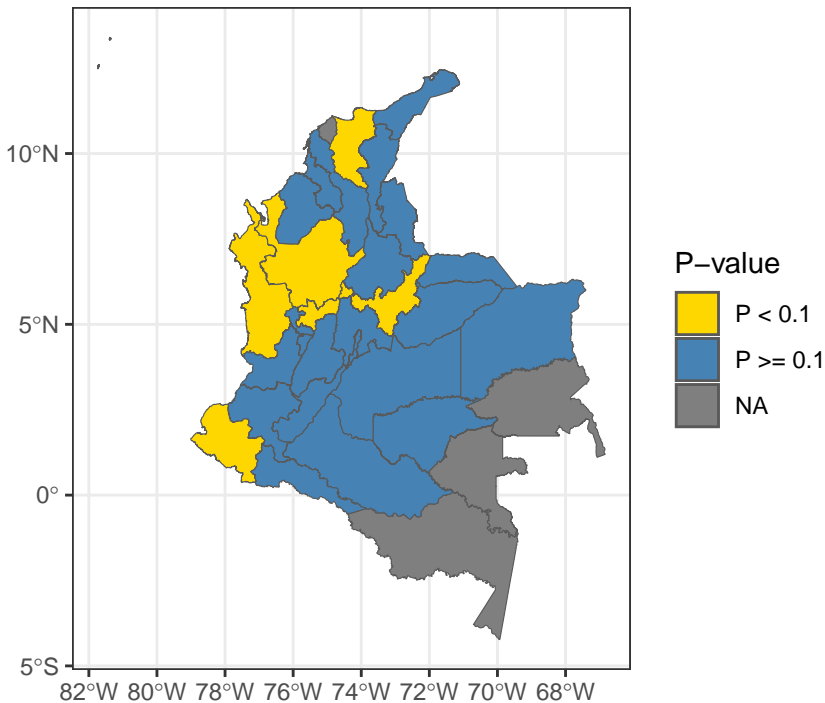

Supplement: Supplementary file 1 — Supplementary Information. [file 41598_2023_48218_MOESM1_ESM.zip › Explaining_conflict_violence_in_terms_of_conflict_actor_dynamics_/figs/departments-1989-2018-p-values.pdf]

# Colombia departments, 2000–2009: P-value

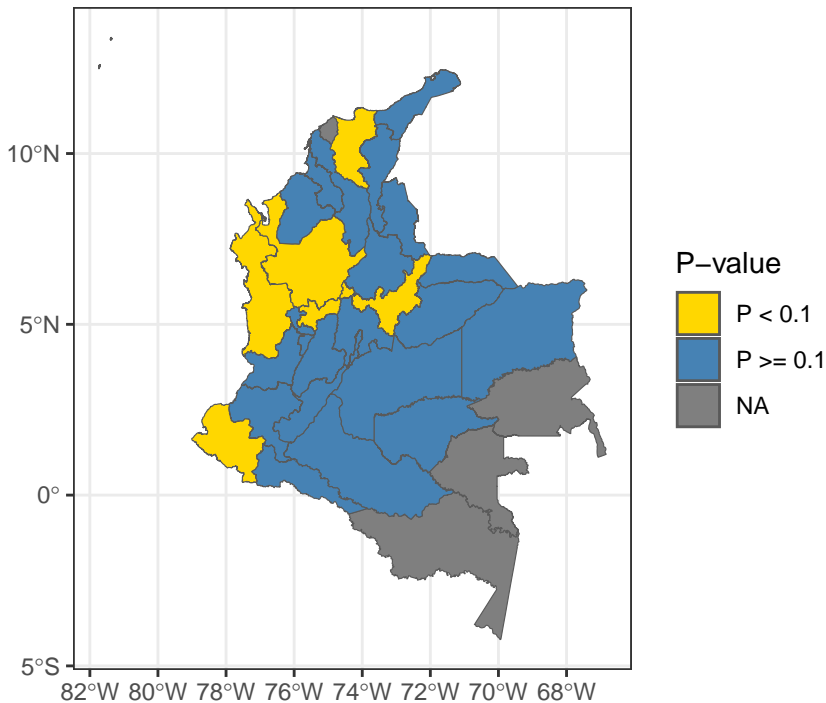

Supplement: Supplementary file 1 — Supplementary Information. [file 41598_2023_48218_MOESM1_ESM.zip › Explaining_conflict_violence_in_terms_of_conflict_actor_dynamics_/figs/departments-2000-2009-p-values.pdf]

# Colombia departments, 2010–2018: P-value

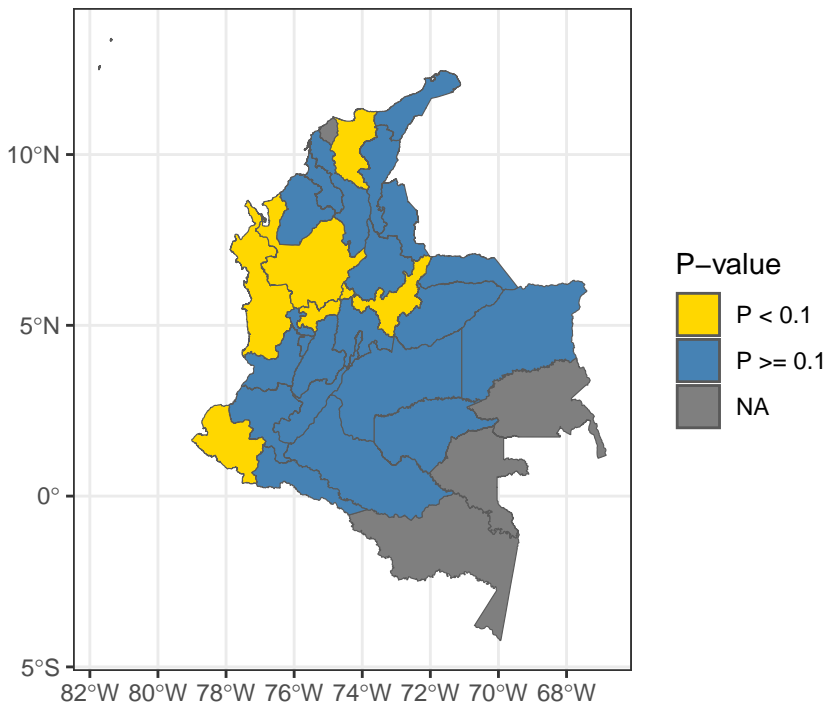

Supplement: Supplementary file 1 — Supplementary Information. [file 41598_2023_48218_MOESM1_ESM.zip › Explaining_conflict_violence_in_terms_of_conflict_actor_dynamics_/figs/departments-2010-2018-p-values.pdf]

# Colombia regions, 1989–1999: Alpha

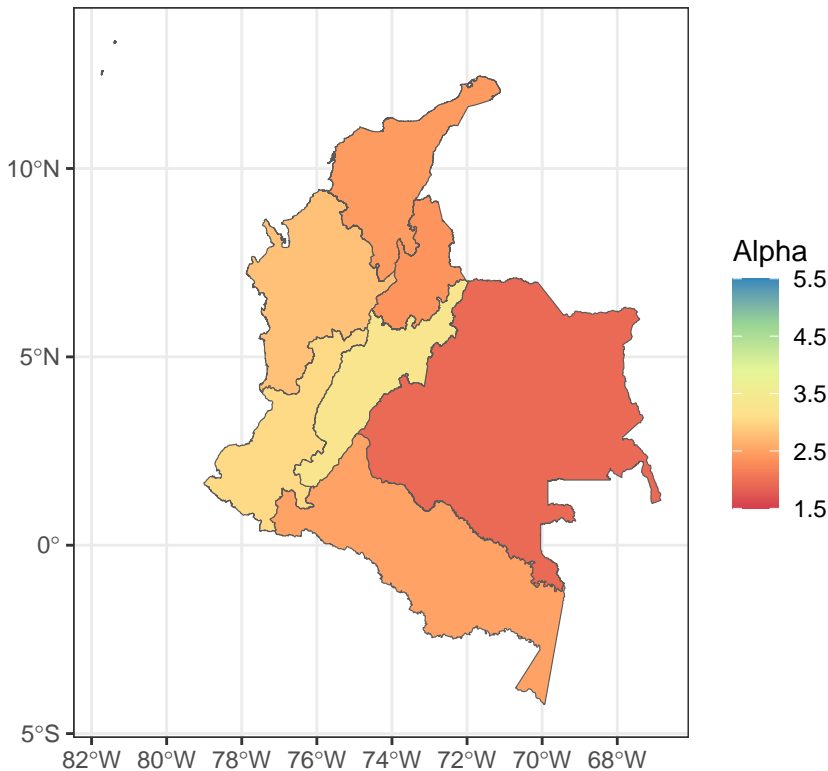

Supplement: Supplementary file 1 — Supplementary Information. [file 41598_2023_48218_MOESM1_ESM.zip › Explaining_conflict_violence_in_terms_of_conflict_actor_dynamics_/figs/regions-1989-1999-alpha.pdf]

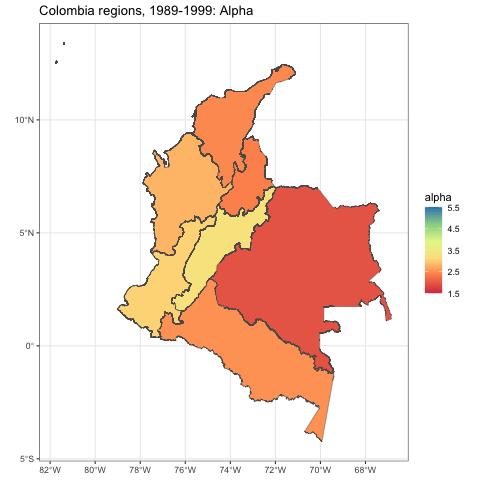

Supplement: Supplementary file 1 — Supplementary Information. [file 41598_2023_48218_MOESM1_ESM.zip › Explaining_conflict_violence_in_terms_of_conflict_actor_dynamics_/figs/regions-1989-1999-alpha.png]

## Colombia regions, 1989–1999: P-value

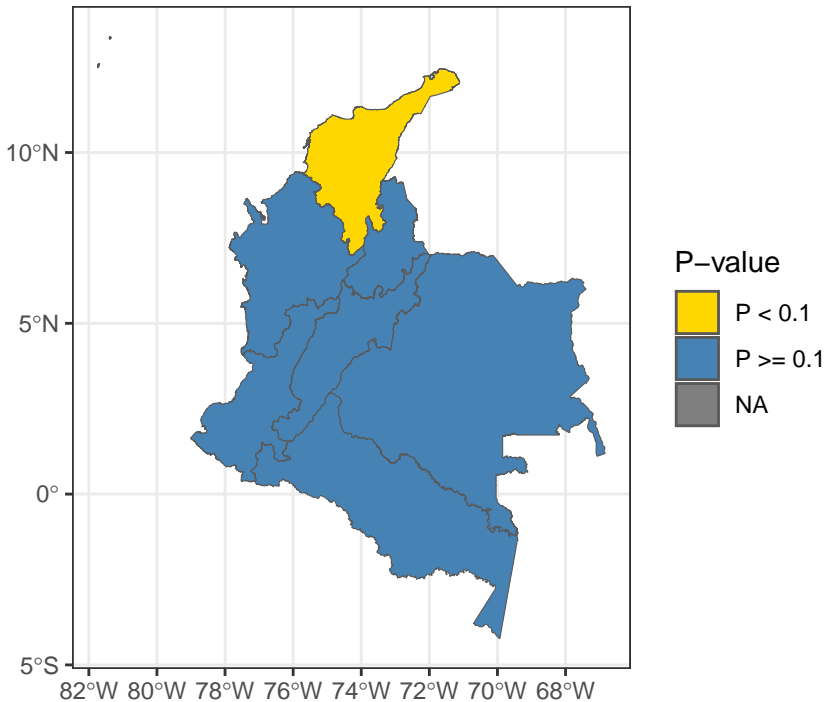

Supplement: Supplementary file 1 — Supplementary Information. [file 41598_2023_48218_MOESM1_ESM.zip › Explaining_conflict_violence_in_terms_of_conflict_actor_dynamics_/figs/regions-1989-1999-p-values.pdf]

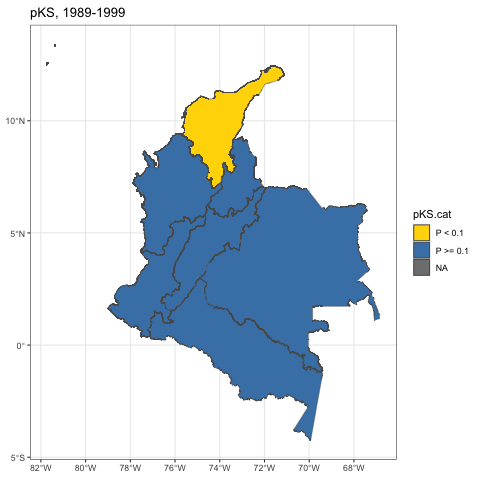

Supplement: Supplementary file 1 — Supplementary Information. [file 41598_2023_48218_MOESM1_ESM.zip › Explaining_conflict_violence_in_terms_of_conflict_actor_dynamics_/figs/regions-1989-1999-p-values.png]

# Colombia regions, 1989–2018: Alpha

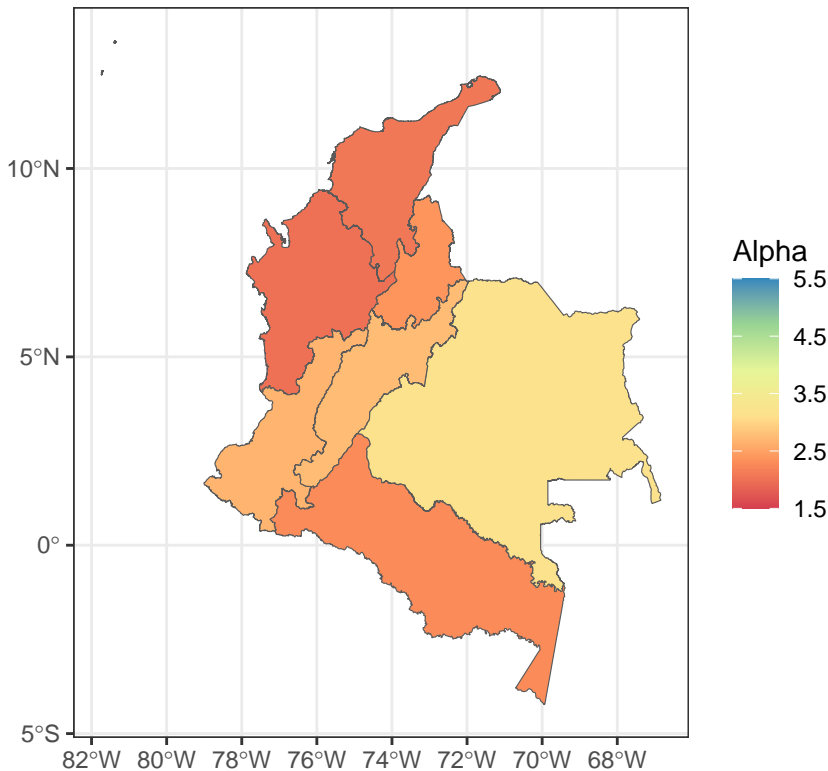

Supplement: Supplementary file 1 — Supplementary Information. [file 41598_2023_48218_MOESM1_ESM.zip › Explaining_conflict_violence_in_terms_of_conflict_actor_dynamics_/figs/regions-1989-2018-alpha.pdf]

# Colombia regions, 1989–2018: P-value

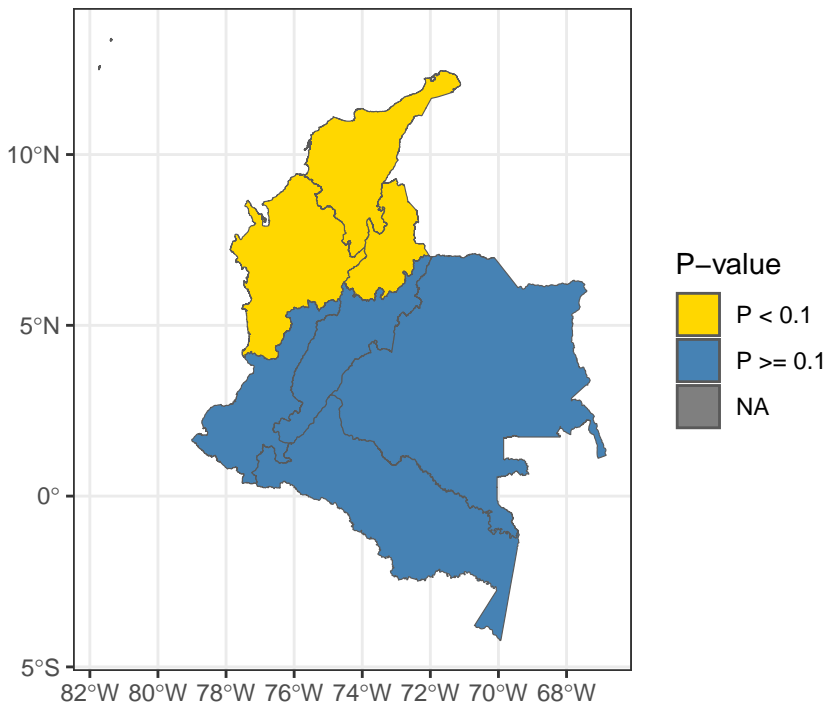

Supplement: Supplementary file 1 — Supplementary Information. [file 41598_2023_48218_MOESM1_ESM.zip › Explaining_conflict_violence_in_terms_of_conflict_actor_dynamics_/figs/regions-1989-2018-p-values.pdf]

# Colombia regions, 2000–2009: Alpha

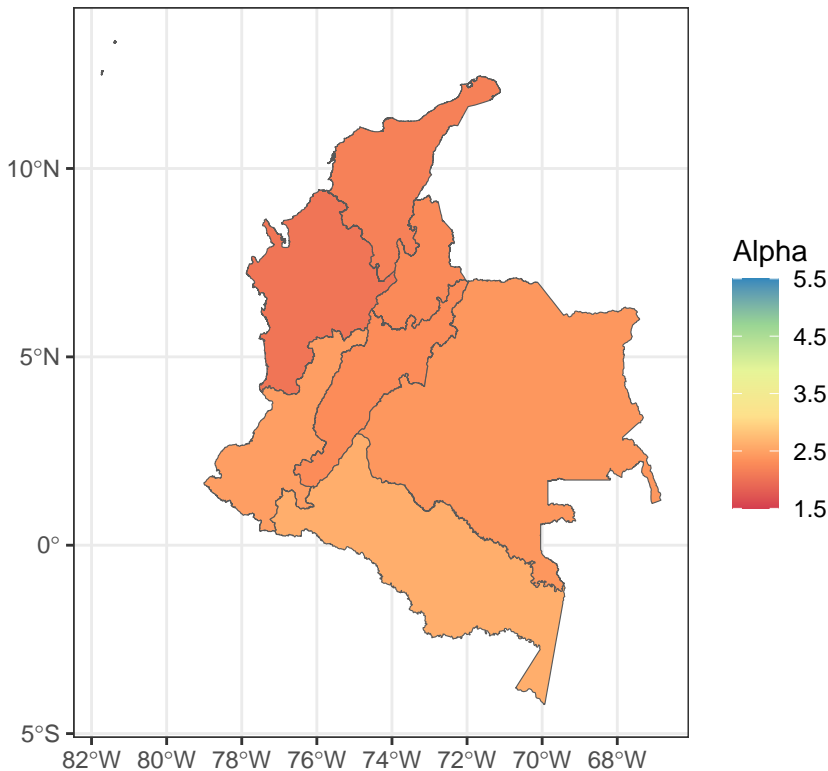

Supplement: Supplementary file 1 — Supplementary Information. [file 41598_2023_48218_MOESM1_ESM.zip › Explaining_conflict_violence_in_terms_of_conflict_actor_dynamics_/figs/regions-2000-2009-alpha.pdf]

# Colombia regions, 2000–2009: P-value

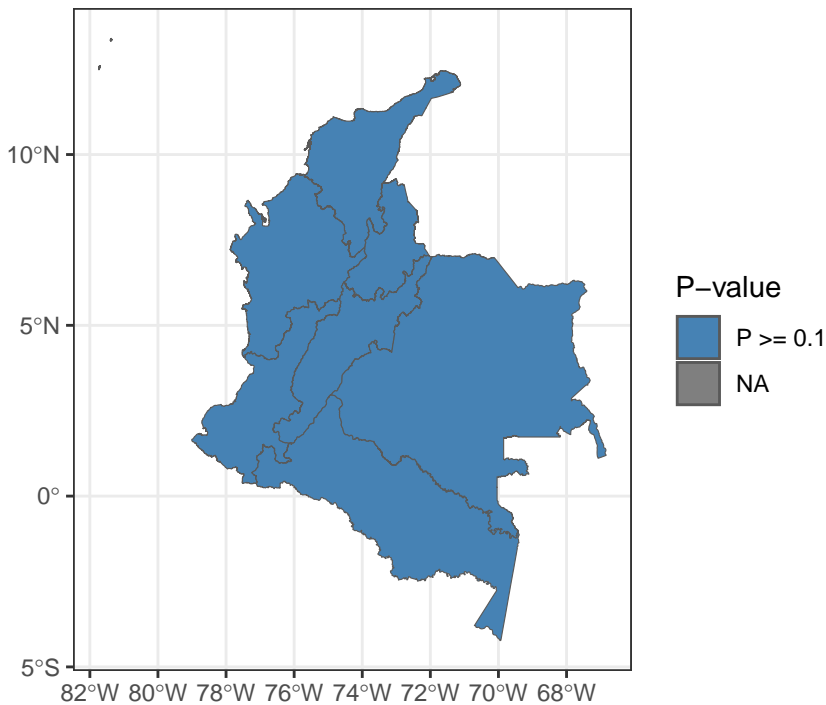

Supplement: Supplementary file 1 — Supplementary Information. [file 41598_2023_48218_MOESM1_ESM.zip › Explaining_conflict_violence_in_terms_of_conflict_actor_dynamics_/figs/regions-2000-2009-p-values.pdf]

Colombia regions, 2010–2018: Alpha

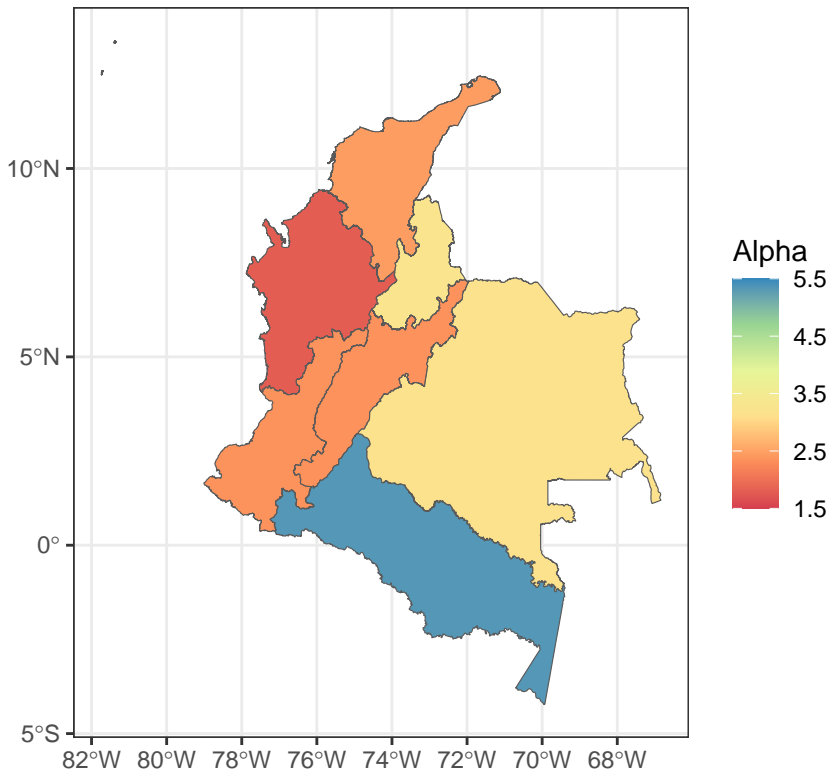

Supplement: Supplementary file 1 — Supplementary Information. [file 41598_2023_48218_MOESM1_ESM.zip › Explaining_conflict_violence_in_terms_of_conflict_actor_dynamics_/figs/regions-2010-2018-alpha.pdf]

## Colombia regions, 2010–2018: P-value

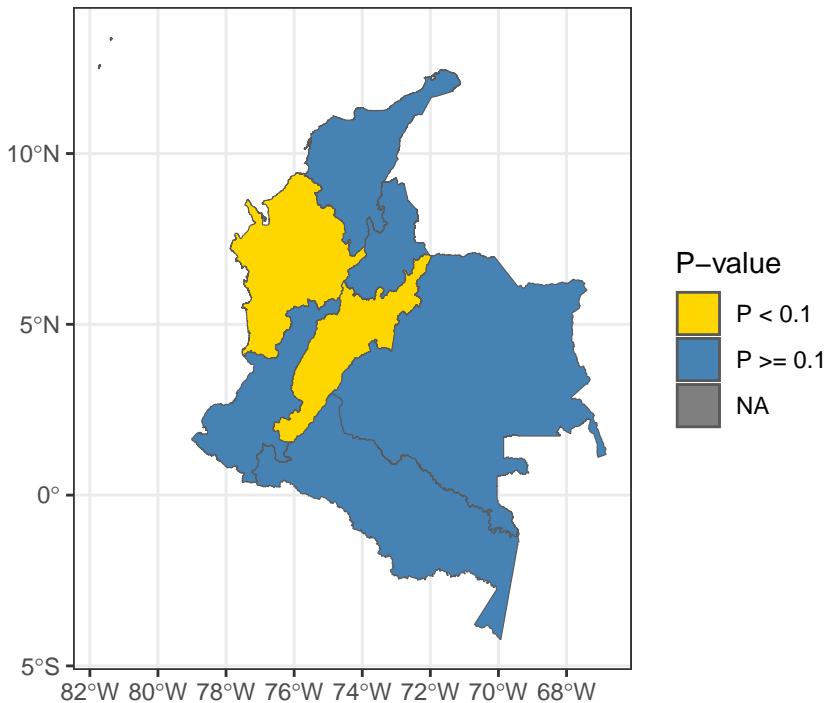

Supplement: Supplementary file 1 — Supplementary Information. [file 41598_2023_48218_MOESM1_ESM.zip › Explaining_conflict_violence_in_terms_of_conflict_actor_dynamics_/figs/regions-2010-2018-p-values.pdf]
